# Options for Final Oocyte Maturation Trigger: Can the Ovarian Response Prediction Index (ORPI) Guide the Optimal Choice?

**DOI:** 10.5935/1518-0557.20250175

**Published:** 2025

**Authors:** Jose G Franco, Claudia G Petersen, Joao Bosco Meziara, Joao Batista A Oliveira

**Affiliations:** 1 Center for Human Reproduction Prof. Franco Jr - Ribeirão Preto, SP, Brazil

**Keywords:** dual trigger, oocyte maturation, ORPI

## Abstract

Dual triggering is recommended in most cases with GnRH antagonists for final oocyte maturation. Although no definitive data support the routine use of dual triggering in normal responders, recent meta-analyses have indicated benefits linked to dual triggering regarding improved reproductive outcomes (oocyte maturation, embryo quality, and clinical pregnancy). Therefore, dual triggering may be considered the preferred trigger for all patients undergoing IVF with antagonist blockade cycles (ORPI in ranges A and B (without hCG dose reduction) and in cases of ORPI in ranges C and D (with hCG dose reduction). Finally, rigorous clinical assessment is essential when an isolated GnRHa trigger is combined with low-dose hCG to correct an inadequate luteal phase. In these cases, the freezing all protocol cannot be ignored.

## INTRODUCTION

The surge of pituitary LH during the mid-menstrual cycle initiates the maturation of the ovum. Elevated plasma LH levels induce cellular changes that drive oocyte maturation and follicle rupture. The final stages of oocyte maturation involve the resumption of meiosis and the transition from metaphase I to metaphase II in oocyte development. In metaphase II, oocytes become capable of being fertilized by sperm. LH binds to its receptors on granulosa cells, inducing the activation of various intracellular signaling pathways, including protein kinase A, protein kinase C, and Ras (a class of proteins known as small GTPases involved in cellular signal transmission). This binding reduces cyclic adenosine monophosphate (cAMP) levels within oocytes, releasing meiosis-promoting factors. In a natural cycle, a surge of FSH also occurs in plasma. Among other functions, the FSH surge ensures an adequate supply of LH receptors on the granulosa layer. It promotes the synthesis of a hyaluronic acid matrix that supports the expansion and dispersion of cumulus cells, allowing the oocyte-cumulus complex to detach and float within the antral fluid (Fritz & Speroff, 2011).

## MEDICATION TRIGGER

Exposure to an LH surrogate is essential for initiating final oocyte maturation during ovarian stimulation cycles for in vitro fertilization (IVF). Chorionic gonadotropin (hCG) and/or GnRH agonists (GnRHa) are commonly utilized for this maturation process. Although both LH and hCG target the same LH receptor, growing evidence indicates that LH has a more pronounced effect on protein kinase B (PKB/AKT) and extracellular signal-regulated protein kinase (ERK1/2) phosphorylation, which is crucial for the proliferation, differentiation, and survival of granulosa cells. Concurrently, hCG enhances intracellular cAMP accumulation, which promotes steroidogenesis. However, selecting the optimal trigger for oocyte maturation also necessitates evaluating the current ovarian response, as the risk of ovarian hyperstimulation syndrome (OHSS) may be closely linked to the most serious adverse trigger choice. In our service, we utilize the ORPI (ovarian response prediction index) along with an analysis of the ovarian response (number of follicles) obtained during the ovarian stimulation cycle to inform our decision on the ideal trigger.

### Calculating the Ovarian Response Prediction Index (ORPI)

The ORPI is calculated by multiplying the AMH (ng/ml) level by the number of antral follicles (2-9 mm), and the result is then divided by the patient’s age (years). This definition of ORPI is based on prior evaluations that found positive correlations between ovarian response to stimulation, AMH levels, and the number of antral follicles while showing a negative correlation with the patient’s age. The derivation of the ORPI is intuitive, stemming from these observed correlations and the testing of different combinations. We aimed for a simple index that is easy to apply in daily practice, combining a limited number of variables whose interactions could enhance the predictive capability of each variable in estimating ovarian response to stimulation (oocyte number expected /Tables 1 and 2) and simultaneously address potential individual deficiencies. The ORPI is defined by the following equation: ORPI = (AMH x AFC)/Patient age (Oliveira *et al*., 2012). The ORPI is directly linked to the quantity of eggs obtained after ovarian stimulation, affecting the risk of OHSS.

**Table 1:** Triggers based on predicted ORPI levels during cycles where follicle rupture was inhibited with a GnRH antagonist or progestogen.

ORPI RANGE	VALUES	OOCYTE(n)	TRIGGER ADVISED
A	<0.25	≤3	hCG (5,000-6,500IU) + GnRHa^*^
B	≥0.25 - <0.9	4-5	hCG (5,000-6,500IU) + GnRHa^*^
C/D	≥0.9 - <1.7	6-14	hCG (2,500-3,250IU) + GnRHa^*^
E	≥1.7	≥16	hCG (1,500IU) + GnRHa^*^ or GnRHa^*^ alone + freezing all

* Usually 0.2-0.3 mg of triptorelin (Gonapeptyl).

**Table 2:** Single trigger with hCG according to ORPI value predictions in cycles in which follicle rupture was inhibited with GnRHa.

ORPI RANGE	VALUES	OOCYTE(n)	TRIGGER ADVISED
A	<0.25	≤3	hCG (5,000-6,500IU)
B	≥0.25 - <0.9	4-5	hCG (5,000-6,500IU)
C/D	≥0.9 - <1.7	6-14	hCG (2,500-3,250IU)
E	≥1.7	≥16	hCG (2,500IU) + freezing all

These precautions are part of our institution’s OHSS zero-tolerance program. Furthermore, in cases of ORPI range E with ovarian volume greater than 150 cm^3^ on days 03 or 05 of fresh embryo transfer, we always choose to freeze all, following protocols established years ago in our center (Petersen *et al*., 2002). Over the last 11 years, there have been no cases of OHSS in our service.

### Single Trigger with hCG (follicle rupture inhibition with GnRHa))

In most cases, hCG has been used to induce final oocyte maturation since the early days of IVF. hCG is a heterodimeric glycoprotein with a high cysteine content and structural similarity to LH, as both proteins share a common subunit and 85% of the amino acid structure of that subunit. Due to their structural similarity, LH and hCG bind to the same LH/hCG receptor (LH-CGR). However, not only do their pharmacokinetics and duration of action differ, but they also induce oocyte maturation through distinct molecular mechanisms. Studies on the plasma half-life of hCG have concluded that it can still be detected in serum approximately 9 to 10 days after intramuscular or subcutaneous administration of 10,000 IU. The plasma clearance of hCG is slow compared to LH, which has a half-life of 1 to 5 hours. Furthermore, the molecular mechanisms of action for LH and hCG in LH-RCG are distinct. In vitro studies have shown that LH primarily promotes granulosa cells’ proliferation, differentiation, and survival by phosphorylating AKT and ERK1/2. At the same time, hCG increases cAMP levels, stimulates steroidogenesis, and leads to progesterone production. OHSS is one of the most serious adverse events associated with hCG triggering. In the pathogenesis of OHSS, an increased number of granulosa cells due to multifollicular growth results in elevated production of vascular endothelial growth factor (VEGF). The slower plasma clearance and longer half-life of hCG enhance the luteinizing stimulus to granulosa cells, thus playing a crucial role in the development of OHSS (Seyhan *et al*., 2013). In GnRHa blockade cycles (Table 2), final oocyte maturation must be achieved with hCG, and the dose reduction will follow the values obtained by ORPI. Finally, a clinical and ultrasound assessment of the ovaries will be crucial in deciding the minimum dose of hCG, whether to perform a fresh transfer or opt for the trigger with hCG followed by freezing all.

### Single Trigger with GnRHa (ORPI RANGE E)

GnRH agonists elicit a more physiological response by triggering surges of LH and FSH and represent a safer option for patients at risk of OHSS when compared to hCG. Gonen *et al*. (1990) assessed the efficacy of a single dose of GnRHa relative to hCG for oocyte maturation. A total of 18 IVF cycles were randomized to receive either a dose of 0.5 mg leuprolide acetate (9 patients) or 5,000 IU hCG (9 patients) as final agents for oocyte maturation. The ovarian stimulation protocol and cycle monitoring were consistent across both groups. Ultimately, the mean number of oocytes retrieved (10±1.4 *versus* 9.7±1.8) and the quality of embryos obtained (2.9±0.1 *versus* 2.4±0.2) showed no significant difference between the two groups. The induced surges of LH and FSH mimic the natural mid-cycle gonadotropin peak. However, a primary concern when using GnRHa to induce final oocyte maturation is the risk of luteal phase insufficiency. LH support is critical for maintaining the corpus luteum during the luteal phase. When GnRHa is used as a trigger, there is a notable reduction in the total amount of endogenous gonadotropins compared to a natural mid-cycle gonadotropin surge. Furthermore, the supraphysiological E2 levels linked to ovulation stimulation protocols exert a negative feedback effect on the hypothalamic-pituitary axis, leading to lower endogenous LH levels during the early and mid-luteal phases (Tavaniotou & Devroey, 2006). Given the hazard of luteal phase insufficiency, Humaidan *et al*. (2005) conducted a randomized study involving 122 patients analyzing the trigger effects of 0.5 mg buserelin (n=55) *versus* 10,000 IU hCG (n=67). The final ovulation maturation induced in the GnRHa group resulted in a significantly higher number of metaphase II (MII) oocytes (*p*<0.02). Additionally, the levels of LH and FSH were substantially higher (*p*<0.001), while levels of progesterone and estradiol were notably lower (*p*<0.001) in the GnRHa group during the luteal phase. The implantation rate were 34% *versus* 3.4% (*p*<0.001), the clinical pregnancy rate was 36% *versus* 6% (*p*=0.002), and the rate of early pregnancy loss was 4% *versus* 79% (*p*= 0.005), all significantly favoring the hCG group. Kolibianakis *et al*. (2005) randomized 106 patients in antagonist-blocked cycles who received either 10,000 IU hCG (n=54) or 0.2mg triptorelin (n=52) as a trigger. There were no significant differences in the number of cumulus-oocyte complexes retrieved, the proportion of metaphase II oocytes, fertilization rates, or the number and quality of embryos transferred between the two groups. However, a significantly lower probability of ongoing pregnancy in the GnRHa group led to the trial’s discontinuation, as per the established stopping rules (odds ratio 0.11; 95%CI, 0.02-0.52). Therefore, a single GnRHa trigger proves to be an effective strategy for improving oocyte recovery in MII and preventing OHSS. Considering these facts, fresh embryo transfers in cycles with an isolated GnRHa trigger necessitate extensive luteal phase support (Singh *et al*., 2023). In conclusion, the all-freezing strategy should always be considered in the clinical evaluation of all cases, as inadequate luteal phase support can lead to lower pregnancy rates in IVF.

### Dual Trigger: standard dose of hCG + GnRHa (ORPI ranges A/B) or a reduced dose of hCG + GnRHa (ORPI ranges C/D)

Given the distinct effects of LH and hCG on receptor signaling, and the physiological gonadotropin surge induced by GnRHa, simultaneous administration of GnRHa and hCG (dual trigger) is recommended for final oocyte maturation. Adding hCG to the GnRHa trigger helps maintain the corpus luteum and aids in oocyte maturation. Shapiro *et al*. (2008) conducted a retrospective study of 45 antagonist cycles in which oocyte maturation was achieved with dual triggering. Patients received leuprolide acetate (4 mg) along with hCG. Individualized hCG dosages were determined based on each patient’s profile and risk factors for OHSS. Oocytes were retrieved 34-36 hours after the trigger and inseminated using intracytoplasmic sperm injection (ICSI); embryos were cultured to the blastocyst stage before transfer. All patients received luteal phase support with E_2_ and progesterone until 8-10 weeks of gestation. In their study, each dual-triggered cycle produced an average of 11 oocytes retrieved; none of the cycles resulted in a miscarriage, and all patients underwent embryo transfer of one or two blastocysts. The early pregnancy loss rate was 17.2% (95% CI, 5.9%-35.8%), while the ongoing pregnancy rate was 53.3% per transfer (95% CI, 37.3%-68.3%). No patients were diagnosed with OHSS, although many had risk factors. One patient with a history of severe OHSS after receiving 5,000 IU hCG in a previous cycle reported no symptoms of OHSS after receiving the dual trigger (2,200 IU hCG and 4 mg leuprolide acetate). Ferraretti *et al.* (2011) analyzed a total of 160 women who met the Bologna criteria for poor ovarian response (POR) and divided them randomly into two groups: group I received 10,000 IU hCG plus 0.2 mg triptorelin, while group II received 10,000 IU hCG only to induce ovulation. The primary outcome measure was the number of oocytes retrieved. Secondary outcome measures included the number of metaphase II oocytes, cancellation rates, the number of embryos retrieved, and chemical and clinical pregnancy rates. Dual trigger was associated with an increase in the number of oocytes retrieved (5.3±1.9 *vs*. 4.5±2.4, *p*=0.014), metaphase II oocytes (3.8±1.4 *vs*. 3.1±1.7, *p*=0.004), and total and grade 1 embryos (2.7±1.1 and 2.3±1.0 *vs*. 1.9±1.2 and 1.1±0.2, *p*=0.001 and 0.021, respectively). Lin *et al*. (2013) retrospectively analyzed 427 IVF cycles where GnRH antagonist was used to block early follicle rupture and observed better reproductive outcomes after fresh embryo transfer in patients with POR who utilized dual trigger. The criteria for defining POR were an AFC of ≤ 5 and a serum AMH of ≤ 1.1 ng/ml. The study group (n=297) was treated with 0.2mg triptorelin plus 6,500IU recombinant hCG, while the control group (n=130) received 6,500 IU recombinant hCG. The dual trigger group exhibited higher oocyte fertilization rates (73.1% *vs*. 58.6%), clinical pregnancy rates (33.0% *vs*. 20.7%), and live birth rates (26.9% *vs*. 14.5%) compared to the single trigger group with hCG. Additionally, the dual trigger group had significantly lower miscarriage rates (17.4% *vs*. 37.0%) and embryo transfer cancellation rates (6.1% *vs*. 15.0%) compared to the control group. The authors concluded that dual triggers could significantly enhance fertilization, clinical pregnancy, and live birth rates in women with POR. Ali *et al*. (2020) evaluated the effectiveness of dual trigger using GnRHa and recombinant human chorionic gonadotropin (rhCG) compared to rhCG alone in normal responders undergoing GnRH antagonist cycles. Study participants were randomized into group I (rhCG group) or II (dual trigger group). The primary outcome measured was the number of mature (MII) oocytes in both groups. Both groups were comparable in terms of baseline demographic and clinical characteristics. Women in the dual trigger group had a statistically significant higher number of retrieved oocytes (*p*=0.001), MII oocytes (*p*=0.01), and the number of grade one embryos (*p*=0.04). Both groups had similar fertilization, implantation, clinical pregnancy, and live birth rates in a fresh cycle. The dual trigger group showed significantly higher rates in clinical pregnancy and live birth after frozen embryo transfer (*p*=0.04, 0.03, respectively). Dual triggering with GnRHa and rHCG improved oocyte maturity and embryo grading for normal responders in GnRH antagonist ICSI cycles. Maged *et al*. (2021) observed in a randomized clinical trial involving patients with POR that a dual trigger is associated with better IVF outcomes than a single trigger with hCG. He *et al*. (2023) conducted a systematic review and meta-analysis of all randomized controlled trials (RCTs) that explored whether dual triggering (GnRHa + hCG) for final oocyte maturation can enhance the number of oocytes retrieved and clinical pregnancy rates in low or normal responders undergoing in vitro fertilization/intracytoplasmic sperm injection (IVF/ICSI) cycles using a GnRH-antagonist protocol. Seven studies were identified, with 898 patients eligible for inclusion in this meta-analysis. The results indicated that the number of oocytes retrieved [WMD = 1.38 (95% CI 0.47-2.28), *I*^2^=66%, *p*=0.003, low evidence], the number of MII oocytes [WMD = 0.7 (95% CI 0.35-1.05), *I*^2^=42%, *p*<0.0001, moderate evidence], the number of embryos [WMD = 0.68 (95% CI 0.07-1.3), *I*^2^=67%, *p*=0.03, low evidence], and the number of good quality embryos [WMD = 1.14 (95% CI 0.35-1.93), *I^2^*=0%, *p*=0.005, moderate evidence] were all significantly higher in the dual trigger group compared to the hCG trigger group. The ovarian response subgroup analysis demonstrated significant differences in all these outcomes for normal responders and no significant differences for low responders, except for the number of MII oocytes. In low responders, clinical pregnancy rates may be enhanced in the dual trigger group [RR = 2.2 (95% CI 1.05-4.61), *I^2^*=28%, *p*=0.04, low evidence]. In conclusion, dual triggering with GnRHa and hCG improved oocyte maturity and embryo grading for normal responders in GnRH-antagonist cycles. Additionally, dual triggering for final oocyte maturation may enhance clinical pregnancy rates in low responders.

### Dual or Double Trigger: low-dose hCG + GnRHa (ORPI RANGE E)

The use of GnRHa alone as a trigger has been recommended for patients at risk of OHSS undergoing ovarian stimulation and prescribed GnRH antagonists to block the early LH surge (ORPI/BAND E). However, a significant loss in clinical results (ongoing pregnancy) has been noted when fresh embryos were transferred, leading clinicians to consider the addition of low dose hCG (1,500 IU) to support the luteal phase. These strategies recommend concurrent administration, either at oocyte retrieval (35 hours after GnRHa) or about five days later. These models address an insufficient luteal phase, but despite reducing the risk of OHSS, they did not eliminate it, similar to using the trigger with GnRHa alone (Seyhan *et al*., 2013). Humaidan *et al*. (2010) proposed administering low-dose hCG (1,500 IU) 35 hours after the induction of final oocyte maturation with a GnRHa trigger during fresh embryo transfers to reduce the rate of ongoing pregnancy loss. One group received a GnRHa trigger (n=152), while the other group received an hCG trigger (n=150), with egg retrieval occurring 34 hours later. A single injection of 1,500 IU hCG was administered intramuscularly 35 hours after GnRHa administration to the first group. The ongoing pregnancy rate in the hCG-only group was 26% compared to 33%; delivery rates were 24% *vs*. 31%; and early pregnancy loss rates were 21% *vs*. 17%, respectively, with no statistical differences between the two groups. Therefore, it can be concluded that a low dose of hCG administered 35 hours after GnRHa benefited the luteal phase from potential luteolysis, resulting in equivalent reproductive outcomes. Due to patient safety and ethical considerations, most studies of dual triggering in high responders at increased risk of OHSS have been retrospective cohort studies. Shapiro *et al*. (2011) conducted a retrospective cohort study of high responders with ≥20 follicles before triggering. They compared the success rates of fresh autologous blastocyst transfers after (a) GnRHa administered with low-dose hCG and standard luteal support (dual trigger), (b) single GnRHa with standard luteal support, and (c) single GnRHa with enhanced luteal support. Their study concluded that ongoing pregnancy rates were significantly higher with dual trigger or enhanced luteal support (57.7% *vs*. 25% *vs*. 50%). One case of OHSS was reported in the dual trigger group and none in the GnRHa trigger group. In another retrospective cohort study (O’Neill *et al*., 2016), the incidence of OHSS, total number, and maturity of oocytes were evaluated in 108 patients with a high ovarian response profile. Group I (n=42) received a GnRHa trigger, while group II (n=66) received a dual trigger with GnRHa + low-dose hCG (1,000 IU). The data indicated a significantly higher incidence of early-onset OHSS after the dual trigger than the GnRHa trigger (8.6% *vs*. 0%). In contrast, the dual trigger was associated with a higher number of total oocytes (OR 1.27; 95% CI 1.18-1.38) and a more significant proportion of mature oocytes (OR 1.10; 95% CI 1.03-1.17) than single GnRH agonist trigger. Although dual or double trigger was linked to a modest increase in oocyte yield, both in quantity and maturity, using dual or double trigger in high responders was associated with an increased risk of OHSS.

## SUMMARY

Dual triggering appears to provide superior outcomes in most GnRH-antagonist cycles, improving oocyte maturation and embryo quality, particularly in normal and poor responders. The ORPI can guide the choice of trigger and appropriate hCG dosing, helping to minimize the risk of OHSS. In high-risk patients (ORPI range E), careful consideration should be given to low-dose hCG supplementation or all-freeze strategies.
